# Navigating ambiguity: A novel neutrosophic cubic shapley normalized weighted Bonferroni Mean aggregation operator with application in the investment environment

**DOI:** 10.1016/j.heliyon.2024.e36781

**Published:** 2024-08-28

**Authors:** Majid Khan, Muhammad Gulistan, Musaed Alhussein, Khursheed Aurangzeb, Adnan Khurshid

**Affiliations:** a Department of Mathematics & Statistics, Hazara University Mansehra, Pakistan; b Department of Electrical and Computer Engineering, University of Alberta, Canada; c Department of Computer Engineering, College of Computer and Information Sciences, King Saud University, P. O. Box 51178, Riyadh, 11543, Saudi Arabia; d College of Economics and Management Zhejiang Normal University, Jinhua, 321004, China

**Keywords:** Neutrosophic cubic set (NCS), Shapley fuzzy measure (SFM), Bonferroni Mean (BM), Decision making (DM)

## Abstract

The Neutrosophic Cubic Shapley Normalized Bonferroni (NC-SNWBM) method represents a cutting-edge approach to decision making theory, combining three distinct mathematical frameworks the neutrosophic cubic sets (NCS), Shapley values, and the Bonferroni aggregation operator. This innovative method addresses the challenges posed by uncertainty, vagueness, and imprecision in decision-making (DM) processes, offering a comprehensive and versatile tool for handling complex and dynamic scenarios. Neutrosophic cubic sets offers a strong platform to handle ambiguous and vague data due to three components Membership Grade (MG), Non-Membership Grade (NMG) and Indeterminancy Grade (IG) in data. By adding Shapley Fuzzy Measures (SFM), which come from cooperative game theory, distribute values among cooperative agents equally and to account for each agent's contributions to all potential coalitions. The Bonferroni aggregation operator—a statistical aggregative tool that regulates the likelihood of many types in error in statistical tests and the interdependence of the input arguments by allowing different values to parameters involved. These values are further improved by normalization in the framework of the NC-SNWBM approach in order to consider the various degrees of impact that agents exert in various circumstances. This operator is smoothly combined with normalized Shapley values and neutrosophic cubic sets in the NC-SNWBM approach to enable the aggregation of data with different levels of imprecision and uncertainty from various sources using NCS. The MG, NMG and IG connected to NCS are important elements of the NC-SNWBM approach. To evaluate each element's contribution to the overall value distribution SFM are used, and the Bonferroni aggregation operator maintains a careful balance between conservatism and significance. Together, these components provide a thorough framework that successfully tackles the problems caused by ambiguity, imprecision, and uncertainty in scenarios involving decision-making. The NC-SNWBM operator is applied to a numerical problem as an application in investment environment and sensitive and comparative analysis are conducted. The recommendation based on sensitive and comparative analysis proposed.

## 1 Introduction

**Fig. 1:**
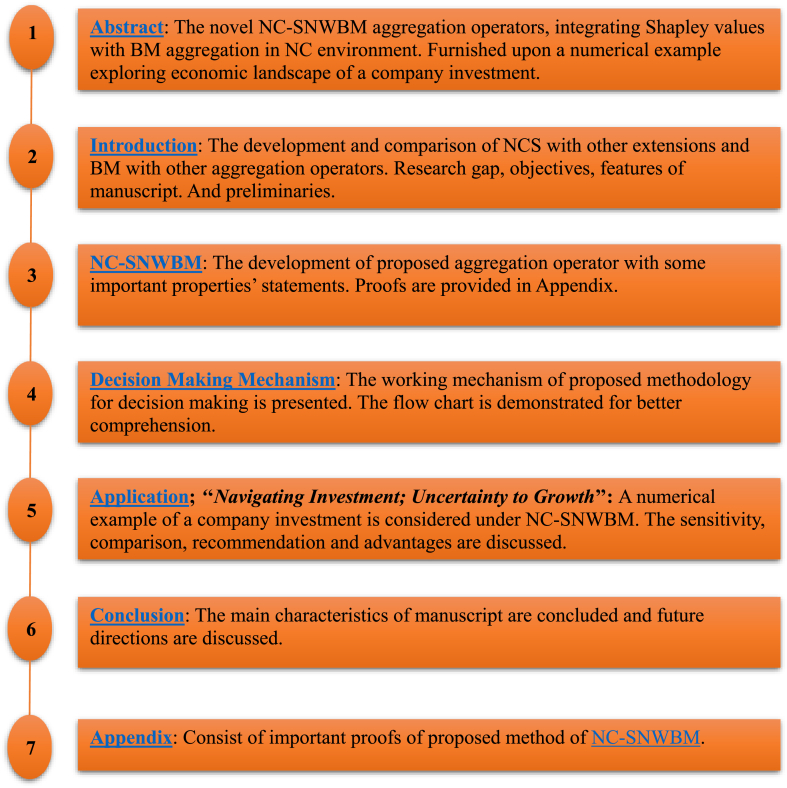
The organization of manuscript.

Organization is missing (before introduction organization is written along with fig 1 in our submitted paper but missing here in this proofread),In an era where DM often involves diverse and incomplete information, a powerful solution is required. To handle diverse and incomplete information different theories have been presented. The Zadeh's fuzzy sets (FS) [1] consisting of Membership Grades (MG) assigning a crisp value from [0,1]. The FS enabled the researchers to handle increasing complexity and uncertainty in decision-making (DM). FS was extended to an interval-valued fuzzy set (IVFS) [2,3], the MG can be assigned as an interval value from [0,1]. Atnassove further extended FS into the intuitionistic fuzzy set (IFS) [4], interval-valued intuitionistic fuzzy set (IVIFS) [5] by instigating NMG in FS; the grades are in interval form in IVIFS. Tora presented the concept of Hesitant Fuzzy Set (HFS) [6] which allows to choose the MG and NMG in the form of sets. The Pythagorean Fuzzy set (PyFS) [7] were presented by taking square roots of MG and NMG. Another extension of FS is the orthopair fuzzy set (OFS) [8], which allow to assign power to MG and NMG. Jun characterized the FS and IVFS as a cubic set (CS) [9], being a hybrid of FS and IVFS can incorporate the information in the crisp and interval form at the same time. These developments put decision-makers in a better position to handle complex and diverse data more efficiently. However, the problem remained the same: as the incomplete frame of situation, the data cannot be completely specified by membership and non-membership grades. The gap can be handled by a picture fuzzy set (PFS) [10]. These extensions of FS contributed significantly in different fields of sciences. A hybrid DM under PFS using Einstein operations [11]. Discussions of algebraic structure in CS [12]. Convexity and capacity in theoretical context [13]. PFS have more practical description using MG, NMG and IG but has limitations that these components are interdependent. This limitation was overcome by neutrosophic set (NS) [14], which consist of MG, NMG, and Indeterminacy Grade (IG) components equation (1). The PFS has the limitations that the components are dependent on, so it proved restriction in choosing these components. On the other hand, NS has no such restriction makes it more versatile and practical. NS was further extended to an interval neutrosophic set (INS) [15] in which MG, NMG and NMG are in the form of interval equation (2). The NS was further extended into Neutrosophic pentapartitioned set (NPS) [16] in which IG has further classified into three more components based on their relevance to MG, NMG and IG. Smrandache presented Plithogenic set and hypersoft set [17,18]. Jun characterized the NS and INS into the neutrosophic cubic set (NS) [19]. The NCS is a handy tool to handle the acceptance, rejection, and neutrality of fuzziness in complex frames of discourse equation (3). These qualities entice the researcher to work in the different fields in recent years. Structuring NC scores and operational laws [20], equations (4), (5), (6), (7), (8), (9). Image completion using segment on NS [21]. Performance indicators renewal energy using NS environment [22]. the Aczel-Alsina aggregation-based outranking method for MADM using NS [23] Evaluation of sports tourism NS in MADM [24]. Dynamic nonlinear simplified NS for MADM [25]. Smart TOPSIS method using NS for green supplier chain [26].

### 1.1 Comparison of different extensions of fuzzy set

The extensions of a fuzzy set consist of some or all components, membership, non-membership, and indeterminate grade, which are fundamental concepts in fuzzy set theory.

**Membership Grade (MG)** The degree that indicates that an element belongs to a fuzzy set is referred to as MG. It shows how much an element complies with the requirements or qualities specified by the set. The values of membership are from 0 to 1, where 1 denotes complete membership and 0 denotes none.

**Non-membership Grade (NMG)**: This denotes the extent to which an element is not a member of a fuzzy set and is the opposite of MG. Additionally, NMG values fall between 0 and 1, where 0 denotes complete membership, and 1 denotes no membership.

**Indeterminate Grade (IG):** The term, which is also used to describe uncertainty or reluctance, describes how unclear or ambiguous it is to give an element in a fuzzy set a definite MG or NGM value. It may be seen as a gauge of fuzziness and indicates the lack of clarity in the decision to become a member or not. When an element's level of MG or NMG is unclear or difficult to assess, an indeterminate grade might help. It permits adaptability and captures the imprecise character of human vision, cognition, and judgment.

These ideas are essential to fuzzy set theory because they offer a framework for handling and modeling uncertainty in a variety of fields where it is challenging to draw clear borders or sharp distinctions. They make it possible to represent and work with ambiguous or fuzzy data, which is helpful in areas like decision analysis, pattern recognition, artificial intelligence, and control systems. It is significant to inscribe that the advantages of extensions of fuzzy sets come with a trade-off with respect to increased computational complexity and the need for more advanced algorithms and methodologies for handling the additional uncertainty. Nonetheless, these extensions offer powerful tools for addressing more complex and uncertain real-world problems compared to typical fuzzy sets.

From Table 1, it can be observed that the three components, membership, non-membership, and indeterminacy provide a strong foundation to handle vagueness, uncertainty, and indeterminacy in data. The PFS and NS have this advantage over other extensions, but in PFS, all the components are interdependent, which makes NS a better choice for researchers. On the other hand, if values assigned to these components are in interval form, it is an easy choice, but crisp values have their own worth. This makes the cubic structure a hybrid of both crisp and interval, a suitable choice. Thus, NCS becomes a more appropriate choice in the extension by having the advantage of independent components and the choice of both crisp and interval forms at the same time Majid et al. [27], designate the novel operation for NCS. The aggregation operators are an imperative part of DM theory. The MCDM problems often implicate conflicting criteria. The aggregation operators are applied to aggregate such conflicting criteria to determine the problems. Gulistan et al. [28], prioritized a MADM using NCS by Einstein aggregations operators. Some important generalization of aggregation operators presented in NCS environment, [29]. The air pollution model was evaluated in NCS environment [30]. A multi-expert approach is applied to unified aggregation operators in NCS [31]. GRA technique for MADM applied in NCS environment [32]. Cosine measure applied to handle DM problems [33].

**Table 1:** The table provides a brief scenario of fuzzy extension.

Set	Advantage	Shortcoming	Ref.
FS	Generalization of the classic set to deal with uncertain data. MG.	The NMG and IG, interval form.	[1]
IVFS	Generalization of FS, Uncertain and vague data, interval form, MG.	The NMG and IG.	[2,3]
IFS	Generalization of FS to deal with uncertain data. MG and NMG	The MG, NMG and IG are interdependent, and interval form.	[4]
IVIFS	Generalization of IFS to deal with uncertain data, MG and NMG.	The MG, NMG and IG are interdependent	[5]
HFS	Generalization of FS and IFS, MG and NMG are is sets form.	IG and MG and NMG are interdependent	[6]
PyFS	Generalization of IFS to deal with uncertain data, MG and NMG, and can assign square root to MG and NMG.	IG and MG and NMG are interdependent, and different exponent then square root.	[7]
CS	Generalization of FS and IVFS to deal with uncertain data. Membership grade in both crisp and interval form	The NMG and IG grades.	[8]
OFS	Generalization of IFS to deal with uncertain data, assign exponent to MG and NMG.	The IG and complexity in computation are due to exponents of MG and NMG.	[9]
PFS	Generalization of IFS to deal with uncertain data. MG, NMG and IG	Interdependent MG, NMG and IG restrict the decision-maker's choices, and this makes computation complicated.	[10]
NS	Generalization of IFS to deal with uncertain data. MG, NMG and IG	Interval values in MG, NMG and IG.	[14]
INS	Generalization of NS to deal with uncertain data. MG, NMG and IG in interval form	Crisps values in MG, NMG and IG.	[15]
NCS	Hybrid of NS and INS. All characteristic mentioned above.		[19]

### 1.2 Comparison of different aggregation operators

The aggregation operators are an imperative component of DM theory. Different aggregation operators are proposed to handle the required landscapes. Handling the complex frame of work need a suffocated operator. The following Table 2, features some important aggregation operators with main characteristics and shortcomings or gaps.

**Table 2:** Comparative analysis of different aggregation analysis.

Aggregation operators	Findings	Gaps	Ref.
Weighted Average (WA)	Provide an easy way of adding weighting technique.	incapable of capturing the intricate DM scenarios, ignored the interrelationships between the input arguments.	[34]
Weighted Geometric (WG)	Provide an easy multiplicative weighting technique. Synthesize ratio assessments in the AHP technique	incapable of capturing the intricate DM scenarios, ignored the interrelationships between the input arguments.	[35]
Ordered weighted average (OWA)	An operator with parameters that provide the value between minimum and maximum. The weights are rearranged according to each input parameter in sequence.	Lake situation to consider hesitant, indeterminate, or bipolar link among the supplied arguments.	[36]
Ordered weighted geometric (OWG)	Extended from WG and OWA		[37]
Einstein	Describe the intuitionistic fuzzy set's Einstein operations in WG and OWG.	ignored the relationships between the input arguments	[38]
Hamacher	In comparison to the algebraic operators and Einstein's t-norm and t-conorm, respectively, Hamacher's t-norm and t-conorm are more generalized and adaptable.	Lake to consider inter relationship between input arguments.	[39]
Prioritized average (PA)	Modeling the significance of the link between the criteria by being aware of their relative relevance and the lack of need for weight vectors	Assume that there is no reciprocal dependence between the input arguments.	[40]
Heronian mean (HM)	identifying the relationships between the combined arguments	ignored the uncertain, erratic, and reluctant information	[41]
Choquet Integral	A generalization of WA and consider the significance of a criterion and interactions between criteria as well.	a generalization form that is applicable to the WA and capable of accounting for the significance of each criterion as well as the interactions among them.	[42]
Bonferroni Mean (BM)	An expansion of geometric and arithmetic meanings Demonstrate how each input argument is interdependent.	did not accurately represent the way decision-makers interacted in general. Not applied in the NCS environment.	[43]

Observing Table 2, the BM [43]operator has the edge over other aggregation operators. So, it can be an important tool to handle complex frames of the environment. However, the shortcoming of BM is over interaction among the Agents or decision-makers, which can be overcome by SFM. The brief description in the literature is as follows. Aggregation operators of an essential component of DM theory. The selection of an appropriate aggregation operator is of key importance in DM. The BM retains the characteristic to handle the interdependence among the input arguments and their coalition. BM was applied to IFS as weighted BM. (WBM) [44], normalized weighted BM (NWBM) [45], partitioned BM (PBM) [46], and geometric BM (GBM) [47]. The limitation of existing BMs is that they are unable to deliberate the contributor contribution and overall interdependence of input arguments.

To achieve long-term success and navigate the complexity in DM, decision makers must make well-informed, strategic decisions. Shapley values provide a fair and balanced method for attributing value among different factors in DM processes by evaluating the contribution of each contributor.

The theory of fuzzy measure [48] plays an important role in DM. This limitation can be handled by involving the Shapley values that provides a fair and balanced method for attributing in DM process by evaluating the contribution of each contributor, stakeholder in the cooperative game [49,50]. In DM, incorporating Shapley values will not only weigh the decision maker, but the individual contribution of a group DM can also be considered [51]. The Shapley divergence were used in VIKOR method of MADM problem [52] The Shapley weighted divergence applied to IFS TODIM [53]. They Shapley values are important featured importance based measured in DM problems [54]. The Shapley values applied to DM in NS environment [55]. Shapley values and normalized Bonferroni operators on PyFS, hesitant bipolar-NS in DM see ([56,57]). DM using induced Shapley Choquet integral in NCS [58].

From this whole discussion it is concluded that NCS is one of better data structuring set due to MG, NMG and IG components and hybrid structure of both crisp and interval value. The BM aggregation operators provides a wide landscape due to generalization of averaging and geometric aggregations and its parameters handling. The SFM are fair values structured as weighted sum of all marginal contributions of contributor and possibilities they make contribution with all possibilities.

**Research Gap** From Table 1, it can be established that NCS is more general set in context of providing both crisp and interval values and all component MG, NMG and IG to enables one express his intuitive more flexibly and comprehensively. Table 2 indicates the superiority of BM over other aggregation methods in Table 2. The citation provides the background of development and combing BM with other aggregation method see (citations (43–47)). The SFM has the characteristics of obtaining a balanced and fair value of individual and interaction with contributors see (citations (48–58)). The BM is not combined with SFM in NCS environment.

**Objectives** Building upon the foundation of NCS, the NC-SNWBM method integrates Shapley values derived from cooperative game theory. Shapley values offer a principled and fair approach to distributing values among cooperative agents based on their marginal contributions to all possible coalitions. By incorporating Shapley values, the NC-SNWBM method ensures that each element's contribution is evaluated within the context of its collaboration with others, fostering fairness in value distribution. Furthermore, the NC-SNWBM method introduces the Bonferroni aggregation operator into this amalgamation of mathematical concepts. The Bonferroni method, known for its role in controlling the family-wise error rate in multiple comparisons, adds a layer of statistical rigor to the aggregation process. This operator balances the need for significance in decision-making with a conservative approach, which is particularly valuable in contexts where the consequences of false positives are critical. One distinctive feature of the NC-SNWBM method lies in the normalization of Shapley values. This additional step ensures that the contributions of individual elements are appropriately scaled, considering the varying degrees of influence exerted in different situations. The normalized Shapley values enhance the method's stability and fairness, making it adaptable to scenarios with dynamic and evolving coalitions. In summary, the Neutrosophic Shapley Normalized Weighted Bonferroni Mean (NC-SNWBM) method stands as an innovative and comprehensive approach to information aggregation. By seamlessly integrating NCS, Shapley values, and the Bonferroni aggregation operator.

**Contribution** This method addresses the intricate challenges of decision-making in the presence of uncertainty, ultimately contributing to a more sophisticated and reliable decision support framework.

The feature contribution of this research:

• Present the SFM, which considers the total interaction between the weights of the criterion.
• Present a new BM operator incorporating SFM in an environment with NCS and suggest an NC-SNWBM operator.
• Application of NC-SNWBM operator in investment-related problems.

### 1.3 Preliminaries

This section consists of some pre-defined definitions and results.

Definition 1 [14] The NS is defined as

(1) $$N=\left\{\left(T_{N}\left(x\right),I_{N}\left(x\right),F_{N}\left(x\right)\right)|x\in X\right\}$$

where $T_{N}\left(x\right),I_{N}\left(x\right),F_{N}\left(x\right)$ are membership, indeterminacy, and non-membership fuzzy functions.

Definition 2 [15] An INS is defined as

(2) $$\tilde{N}=\left\{\left({\tilde{T}}_{N}\left(x\right),{\tilde{I}}_{N}\left(x\right),{\tilde{F}}_{N}\left(x\right)\right)|x\in X\right\}$$

where ${\tilde{T}}_{N}\left(x\right),{\tilde{I}}_{N}\left(x\right),{\tilde{F}}_{N}\left(x\right)$, are interval valued fuzzy membership, indeterminacy, and non-membership function.

Definition 3 [19] An NCS defined as

(3) $$Nc=\left\{\left({\tilde{T}}_{N}\left(x\right),{\tilde{I}}_{N}\left(x\right),{\tilde{F}}_{N}\left(x\right),T_{N}\left(x\right),I_{N}\left(x\right),F_{N}\left(x\right)\right)|x\in \Gamma \right\}$$

where $\left({\tilde{T}}_{N}\left(x\right)=\left[T_{N}^{L}\left(x\right),T_{N}^{U}\left(x\right)\right],{\tilde{I}}_{N}\left(x\right)=\left[I_{N}^{L}\left(x\right),I_{N}^{U}\left(x\right)\right],{\tilde{F}}_{N}\left(x\right)=\left[F_{N}^{L}\left(x\right),F_{N}^{U}\left(x\right)\right]\right)$ is an INS and $\left(T_{N}\left(x\right),I_{N}\left(x\right),F_{N}\left(x\right)\right)$ is an NS. Where $0\leq T_{N}+I_{N}+F_{N}\leq 3,\tilde{0}\leq {\tilde{T}}_{N},{\tilde{I}}_{N},{\tilde{F}}_{N}\leq \tilde{3}$.

For convenience, an NCS written as $Nc=\left(\left[T_{N}^{L},T_{N}^{U}\right],\left[I_{N}^{L},I_{N}^{U}\right],\left[F_{N}^{L},F_{N}^{U}\right],T_{N},I_{N},F_{N}\right)$.

Definition 4 [27] Let $N=\left(\left[T_{N}^{L},T_{N}^{U}\right],\left[I_{N}^{L},I_{N}^{U}\right],\left[F_{N}^{L},F_{N}^{U}\right],T_{N},I_{N},F_{N}\right)$, $M=\left(\left[T_{M}^{L},T_{M}^{U}\right],\left[I_{M}^{L},I_{M}^{U}\right],\left[F_{M}^{L},F_{M}^{U}\right],T_{M},I_{M},F_{M}\right)$ and η be a scalar, then

(4) $$N⊕M=\left(\begin{array}{c} \left[T_{N}^{L}+T_{M}^{L}-T_{N}^{L}T_{M}^{L},T_{N}^{U}+T_{M}^{U}-T_{N}^{U}T_{M}^{U}\right],\left[I_{N}^{L}+I_{M}^{L}-I_{N}^{L}I_{M}^{L},I_{N}^{U}+I_{M}^{U}-I_{N}^{U}I_{M}^{U}\right],\\ \left[F_{N}^{L}F_{M}^{L},F_{N}^{U}F_{M}^{U}\right],T_{N}T_{M},I_{N}I_{M},F_{N}+F_{M}-F_{N}F_{M} \end{array}\right)$$

(5) $$N⊗M=\left(\begin{array}{c} \left[T_{N}^{L}T_{M}^{L},T_{N}^{U}T_{M}^{U}\right],\left[I_{N}^{L}I_{M}^{L},I_{N}^{U}I_{M}^{U}\right],\left[F_{N}^{L}+F_{M}^{L}-F_{N}^{L}F_{M}^{L},F_{N}^{U}+F_{M}^{U}-F_{N}^{U}F_{M}^{U}\right],\\ \;T_{N}+T_{M}-T_{N}T_{M},I_{N}+I_{M}-I_{N}I_{M},F_{N}F_{M} \end{array}\right)$$

(6) $$\eta N=\left(\begin{array}{c} \left[1-{\left(1-T_{N}^{L}\right)}^{\eta },1-{\left(1-T_{N}^{U}\right)}^{\eta }\right],\left[1-{\left(1-I_{N}^{L}\right)}^{\eta },1-{\left(1-I_{N}^{U}\right)}^{\eta }\right],\\ \left[{\left(F_{N}^{L}\right)}^{\eta },{\left(F_{N}^{U}\right)}^{\eta }\right],{\left(T_{N}\right)}^{\eta },{\left(I_{N}\right)}^{\eta },1-{\left(1-F_{N}\right)}^{\eta } \end{array}\right)$$

(7) $$N^{\eta }=\left(\begin{array}{c} \left[{\left(T_{N}^{L}\right)}^{\eta },{\left(T_{N}^{U}\right)}^{\eta }\right],\left[{\left(I_{N}^{L}\right)}^{\eta },{\left(I_{N}^{U}\right)}^{\eta }\right],\left[1-{\left(1-F_{N}^{L}\right)}^{\eta },1-{\left(1-F_{N}^{U}\right)}^{\eta }\right],\\ \;1-{\left(1-T_{N}\right)}^{\eta },1-{\left(1-I_{N}\right)}^{\eta },{\left(F_{N}\right)}^{\eta } \end{array}\right)$$

(8) $$Score\left(N\right)=T_{N}^{U}-F_{N}^{U}+T_{N}^{L}\;-F_{N}^{L}+T_{N}-F_{N}$$

(9) $$Accuracy\left(N\right)=\left(T_{N}^{L}+T_{N}^{U}+I_{N}^{L}+I_{N}^{U}+F_{N}^{L}+F_{N}^{U}+T_{N}+I_{N}+F_{N}\right)/9$$

In the following definition the SFM equation (10) and generalized SFM equation (11) are defined.

Definition 7 [48] Let $Њ=\left\{{Б}_{1},{Б}_{2},{Б}_{3},\ldots ,{Б}_{\ddot{n}}\right\}$ be a set. a fuzzy measure θ on $\overset{⌢}{T}$ is defined as a function θ:Њ→[0,1] fulfilling the following properties.

i. θ(ϕ)=0andθ(Њ)=1.
ii. For ${Б}_{1},{Б}_{2}\in P\left(Њ\right),{Б}_{1}\subseteq {Б}_{2}\Rightarrow \theta \left({Б}_{1}\right)\leq \theta \left({Б}_{2}\right)$ where P(Њ) is power set of Њ.

Definition 8 [48] In MCDM for ${Б}_{1},{Б}_{2}\in P\left(Њ\right)$ such that ${Б}_{1}\cap {Б}_{2}=\phi$, three type of interactive relation are possible that is

**Additive measure:** if ${Б}_{1},{Б}_{2}$ are independent, then $\theta \left({Б}_{1}\cup {Б}_{2}\right)=\theta \left({Б}_{1}\right)+\theta \left({Б}_{2}\right)$.

**Super additive measure:** If ${Б}_{1},{Б}_{2}$ are positive synergetic interaction, then $\theta \left({Б}_{1}\cup {Б}_{2}\right)>\theta \left({Б}_{1}\right)+\theta \left({Б}_{2}\right)$.

**Sub additive measure:** If ${Б}_{1},{Б}_{2}$ are negative synergetic interaction, then $\theta \left({Б}_{1}\cup {Б}_{2}\right)<\theta \left({Б}_{1}\right)+\theta \left({Б}_{2}\right)$.

Definition 5 [49] **T**he Shapley value $\varpi_{S}^{Sh}\left(\theta ,N\right)$ of a contributor is a weighted averaging value of the marginal contribution θ(U∪V)−θ(V) of contributor with all combinations, is defined as:

(10) $$\varpi_{S}^{Sh}\left(\theta ,N\right)=\sum_{U\subseteq N\backslash S}\frac{\left(\ddot{n}-u-1\right)!u!}{\left(\ddot{n}\right)!}\left(\theta \left(U\cup V\right)-\theta \left(V\right)\right),\forall U\subseteq N$$

where θ is FM λ, on N, and the cardinality of U,N and V is respectively u, $\ddot{n}$ and v.

Here, $\frac{\left(\ddot{n}-u-1\right)!u!}{\left(\ddot{n}\right)!}$, is weight and θ(U∪V)−θ(V) is marginal contribution of contributor U with coalition V. This make Shapley a fair value in each coalition because it is weighted sum of all marginal contribution of contributor and probability they make contribution and then sum of all possibilities.

Definition 6 [50] Generalized Shapley value of a contributor is defined as:

(11) $$\varpi_{S}^{Sh}\left(\theta_{\lambda },N\right)=\sum_{U\subseteq N\backslash S}\frac{\left(\ddot{n}-u-v\right)!v!}{\left(\ddot{n}-u+1\right)!}\left(\theta_{\lambda }\left(U\cup V\right)-\theta_{\lambda }\left(V\right)\right),\forall U\subseteq N$$

where $\theta_{\lambda }-$ FM expressed as

$$\theta_{\lambda }\left(A\right)=\left\{ \begin{array}{c} \frac{1}{\lambda }\left(\prod_{k\in \overset{⌢}{T}}\left(\left(\lambda \theta_{\lambda }\left(i\right)+1\right)-1\right)\right)\;if\lambda \neq 0\\ \sum_{k\in \overset{⌢}{T}}\lambda \theta_{\lambda }\left(k\right)\;if\lambda =0 \end{array}\right.$$

where $\prod_{c\in N}\left(1+\theta_{\lambda }\left\{c_{k}\right\}\right)=1+\theta_{\lambda }$ is used to measure λ.

## 2 Neutrosophic cubic shapley normalized weighted Bonferroni Mean operator

This section consists of neutrosophic cubic shapley normalized weighted Bonferroni Mean (NC-SNWBM) operators and some important results.

Definition Let $N_{r}=\left(\left[{\left(T_{N}\right)}_{r}^{L},{\left(T_{N}\right)}_{r}^{U}\right],\left[{\left(I_{N}\right)}_{r}^{L},{\left(I_{N}\right)}_{r}^{U}\right],\left[{\left(F_{N}\right)}_{r}^{L},{\left(F_{N}\right)}_{r}^{U}\right],{\left(T_{N}\right)}_{r},{\left(I_{N}\right)}_{r},{\left(F_{N}\right)}_{r}\right)$ and $N_{s}=\left(\left[{\left(T_{N}\right)}_{s}^{L},{\left(T_{N}\right)}_{s}^{U}\right],\left[{\left(I_{N}\right)}_{s}^{L},{\left(I_{N}\right)}_{s}^{U}\right],\left[{\left(F_{N}\right)}_{s}^{L},{\left(F_{N}\right)}_{s}^{U}\right],{\left(T_{N}\right)}_{s},{\left(I_{N}\right)}_{s},{\left(F_{N}\right)}_{s}\right)$, where $\left(1\leq r,s\leq \ddot{n}\right)$ be a the collection of NC values, and p,q≥0 then the neutrosophic cubic BM (NC-BM) operator is defined as:

$$NC-BM\left(N\right)={\left(\frac{1}{n\left(n-1\right)}\sum_{\begin{array}{c} r,s=1\\ r\neq s \end{array}}^{n}{\left(N_{r}\right)}^{p}⊗{\left(N_{s}\right)}^{q}\right)}^{\frac{1}{p+q}}$$

The neutrosophic cubic normalized Weighted BM(NC-NWBM) operator is defined as:

$NC-NWBM\left(N\right)={\left(\frac{\varpi_{r}\varpi_{s}}{1-\varpi_{r}}\sum_{\begin{array}{l} r,s=1\\ r\neq s \end{array}}^{n}{\left(N_{r}\right)}^{p}⊗{\left(N_{s}\right)}^{q}\right)}^{\frac{1}{p+q}}$ where $\varpi_{r}$, $\varpi_{r}$ are the corresponding weight of arguments such that $\varpi_{r},\varpi_{r}\in \left[0,1\right]$ and $\sum_{r=1}^{n}\varpi_{r}=1$, $\sum_{s=1}^{n}\varpi_{s}=1$.

The neutrosophic cubic Shapley Normalized Weighted BM(NC-SNWBM) operator is defined as:

$NC-SNWBM\left(N\right)={\left(\sum_{\begin{array}{l} r,s=1\\ r\neq s \end{array}}^{n}\frac{\varpi_{r}^{Sh}\left(\mu ,N\right)\varpi_{s}^{Sh}\left(\mu ,N\right)}{1-\varpi_{r}^{Sh}\left(\mu ,N\right)}\left({\left(N_{r}\right)}^{p}⊗{\left(N_{s}\right)}^{q}\right)\right)}^{\frac{1}{p+q}}$, where $\varpi_{r}^{Sh}\left(\mu ,N\right)$ is SFM of $N_{r}$ and $\varpi_{s}^{Sh}\left(\mu ,N\right)$ is SFM of $N_{s}$ with $\sum_{r=1}^{n}\varpi_{r}^{Sh}\left(\mu ,N\right)=1,\sum_{s=1}^{n}\varpi_{s}^{Sh}\left(\mu ,N\right)=1$ for $\left(1\leq r,s\leq \ddot{n}\right)$ respectively.

Some important theorem are presented for NC-SNWBM aggregation operator.

Theorem 1 (*Equality*).

$$NC-SNWBM\left(N\right)=\left(\begin{array}{c} \left[{\left(1-\overset{n}{\underset{\begin{array}{c} r,s=1\\ r\neq s \end{array}}{\Pi }}{\left(1-\left({\left({\left(T_{N}\right)}_{r}^{L}\right)}^{p}⊗{\left({\left(T_{N}\right)}_{s}^{L}\right)}^{q}\right)\right)}^{\frac{\varpi_{r}\varpi_{s}}{1-\varpi_{s}}}\right)}^{\frac{1}{p+q}},{\left(1-\overset{n}{\underset{\begin{array}{c} r,s=1\\ r\neq s \end{array}}{\Pi }}{\left(1-\left({\left({\left(T_{N}\right)}_{r}^{U}\right)}^{p}⊗{\left({\left(T_{N}\right)}_{s}^{U}\right)}^{q}\right)\right)}^{\frac{\varpi_{r}\varpi_{s}}{1-\varpi_{s}}}\right)}^{\frac{1}{p+q}}\right],\\ \left[{\left(1-\overset{n}{\underset{\begin{array}{c} r,s=1\\ r\neq s \end{array}}{\Pi }}{\left(1-\left({\left({\left(I_{N}\right)}_{r}^{L}\right)}^{p}⊗{\left({\left(I_{N}\right)}_{s}^{L}\right)}^{q}\right)\right)}^{\frac{\varpi_{r}\varpi_{s}}{1-\varpi_{s}}}\right)}^{\frac{1}{p+q}},{\left(1-\overset{n}{\underset{\begin{array}{c} r,s=1\\ r\neq s \end{array}}{\Pi }}{\left(1-\left({\left({\left(I_{\varsigma }\right)}_{r}^{U}\right)}^{p}⊗{\left({\left(I_{N}\right)}_{s}^{U}\right)}^{q}\right)\right)}^{\frac{\varpi_{r}\varpi_{s}}{1-\varpi_{s}}}\right)}^{\frac{1}{p+q}}\right],\\ \left[1-{\left(1-\overset{n}{\underset{\begin{array}{c} r,s=1\\ r\neq s \end{array}}{\Pi }}{\left(1-\left({\left({\left(1-F_{N}\right)}_{r}^{L}\right)}^{p}⊗{\left({\left(1-F_{N}\right)}_{s}^{L}\right)}^{q}\right)\right)}^{\frac{\varpi_{r}\varpi_{s}}{1-\varpi_{s}}}\right)}^{\frac{1}{p+q}},1-{\left(1-\overset{n}{\underset{\begin{array}{c} r,s=1\\ r\neq s \end{array}}{\Pi }}{\left(1-\left({\left({\left(1-F_{N}\right)}_{r}^{U}\right)}^{p}⊗{\left({\left(1-F_{N}\right)}_{s}^{U}\right)}^{q}\right)\right)}^{\frac{\varpi_{r}\varpi_{s}}{1-\varpi_{s}}}\right)}^{\frac{1}{p+q}}\right],\\ 1-{\left(1-\overset{n}{\underset{\begin{array}{c} r,s=1\\ r\neq s \end{array}}{\Pi }}{\left(1-\left({\left({\left(1-T_{N}\right)}_{r}\right)}^{p}⊗{\left({\left(1-T_{N}\right)}_{s}\right)}^{q}\right)\right)}^{\frac{\varpi_{r}\varpi_{s}}{1-\varpi_{s}}}\right)}^{\frac{1}{p+q}},1-{\left(1-\overset{n}{\underset{\begin{array}{c} r,s=1\\ r\neq s \end{array}}{\Pi }}{\left(1-\left({\left({\left(1-I_{N}\right)}_{r}\right)}^{p}⊗{\left({\left(1-I_{N}\right)}_{s}\right)}^{q}\right)\right)}^{\frac{\varpi_{r}\varpi_{s}}{1-\varpi_{s}}}\right)}^{\frac{1}{p+q}},\\ {\left(1-\overset{n}{\underset{\begin{array}{c} r,s=1\\ r\neq s \end{array}}{\Pi }}{\left(1-\left({\left({\left(F_{N}\right)}_{r}\right)}^{p}⊗{\left({\left(F_{N}\right)}_{s}\right)}^{q}\right)\right)}^{\frac{\varpi_{r}\varpi_{s}}{1-\varpi_{s}}}\right)}^{\frac{1}{p+q}} \end{array}\right)$$

Next, the important algebraic properties of NC-SNWBM are investigated.

Theorem 2 (Reducibility) If $\varpi_{r}\left(\mu ,N\right)=\left(\frac{1}{n},\frac{1}{n},\ldots ,\frac{1}{n}\right)\text{,}$ then $\text{NC}-\text{SNWBM}\left(\varsigma_{r}\right)=\text{NC}-\text{BM}\left(\varsigma \right)$

Theorem 3 (Idempotency) Let $N_{r}=\left(\left[{\left(T_{N}\right)}_{r}^{L},{\left(T_{N}\right)}_{r}^{U}\right],\left[{\left(I_{N}\right)}_{r}^{L},{\left(I_{N}\right)}_{r}^{U}\right],\left[{\left(F_{N}\right)}_{r}^{L},{\left(F_{N}\right)}_{r}^{U}\right],{\left(T_{N}\right)}_{r},{\left(I_{N}\right)}_{r},{\left(F_{N}\right)}_{r}\right)$ for $\left(1\leq r\leq \ddot{n}\right)$ be a set of NCs and are all equal, i.e., $N_{r}=N$ for all r. Then, $\text{NC}-\text{SNWBM}\left(N_{r}\right)=N$

Theorem 4 (Monotonicity) Let $N_{r}=\left(\left[{\left(T_{N}\right)}_{r}^{L},{\left(T_{N}\right)}_{r}^{U}\right],\left[{\left(I_{N}\right)}_{r}^{L},{\left(I_{N}\right)}_{r}^{U}\right],\left[{\left(F_{N}\right)}_{r}^{L},{\left(F_{N}\right)}_{r}^{U}\right],{\left(T_{N}\right)}_{r},{\left(I_{N}\right)}_{r},{\left(F_{N}\right)}_{r}\right)$ and $N_{s}=\left(\left[{\left(T_{N}\right)}_{s}^{L},{\left(T_{N}\right)}_{s}^{U}\right],\left[{\left(I_{N}\right)}_{s}^{L},{\left(I_{N}\right)}_{s}^{U}\right],\left[{\left(F_{N}\right)}_{s}^{L},{\left(F_{N}\right)}_{s}^{U}\right],{\left(T_{N}\right)}_{s},{\left(I_{N}\right)}_{s},{\left(F_{N}\right)}_{s}\right)$ , where $\left(1\leq r,s\leq \ddot{n}\right)$ be a the collection of NC values. If $N_{r}\leq N_{s}$ then $\text{NC}-\text{SNWBM}\left(N_{r}\right)\leq \text{NC}-\text{SNWBM}\left(N_{r}*\right)$

Theorem 5 (Boundedness) Let $N_{r}=\left(\left[{\left(T_{N}\right)}_{r}^{L},{\left(T_{N}\right)}_{r}^{U}\right],\left[{\left(I_{N}\right)}_{r}^{L},{\left(I_{N}\right)}_{r}^{U}\right],\left[{\left(F_{N}\right)}_{r}^{L},{\left(F_{N}\right)}_{r}^{U}\right],{\left(T_{N}\right)}_{r},{\left(I_{N}\right)}_{r},{\left(F_{N}\right)}_{r}\right)$

for $\left(1\leq r\leq \ddot{n}\right)$ be NCs and ${\left(N_{r}\right)}^{-}=\left(\left[\min\;{\left(T_{N}\right)}_{r}^{L},\min\;{\left(T_{N}\right)}_{r}^{U}\right],\left[\min\;{\left(I_{N}\right)}_{r}^{L},\min\;{\left(I_{N}\right)}_{r}^{U}\right],\left[1-\max\;{\left(F_{N}\right)}_{r}^{L},1-\max\;{\left(F_{N}\right)}_{r}^{L}\right],\min\;{\left(T_{N}\right)}_{r},\min\;{\left(I_{N}\right)}_{r},1-\max\;{\left(F_{N}\right)}_{r}\right)$,

${\left(N_{r}\right)}^{+}=\left(\left[\max\;{\left(T_{N}\right)}_{r}^{L},\max\;{\left(T_{N}\right)}_{r}^{U}\right],\left[\max\;{\left(I_{N}\right)}_{r}^{L},\max\;{\left(I_{N}\right)}_{r}^{U}\right],\left[1-\min\;{\left(F_{N}\right)}_{r}^{L},1-\min\;{\left(F_{N}\right)}_{r}^{L}\right],\max\;{\left(T_{N}\right)}_{r},\max\;{\left(I_{N}\right)}_{r},1-\min\;{\left(F_{N}\right)}_{r}\right)$.

Then ${\left(N_{r}\right)}^{-}\leq \text{NC}-\text{SNWBM}\left(N_{r}\right)\leq {\left(N_{r}\right)}^{+}$.

The proof of theorems are provided in appendix.

Some special cases of NC – SNWBM operator is discussed below:

It is worth mentioning that if weights are considered in the form of SFM the operators reduced to SNW otherwise to weighted aggregations operators.

Case 1 If p≠0qndq=0(p=0qndq≠0), then NC-SNWBM operator reduces as follows:

$\text{NC}-\text{SNWBM}\left(N\right)={\left(\sum_{\begin{array}{c} r,s=1\\ r\neq s \end{array}}^{n}\varpi_{r}^{\text{Sh}}\left(\mu ,N\right){\left(N_{r}\right)}^{p}\right)}^{\frac{1}{p}}$, is prioritized power, NC-SNWBM.

• If p = 1, NC-SNWBM reduces to NC-SNWA.
• If p = 2, NC-SNWBM reduces to quadratic NC-SNWA, so on.

Case 2 If p=q, then NC-SNWBM operator reduces as follows:

$$NC-SNWBM\left(N\right)={\left(\sum_{\begin{array}{c} r,s=1\\ r\neq s \end{array}}^{n}\frac{\varpi_{r}^{Sh}\left(\mu ,N\right)\varpi_{s}^{Sh}\left(\mu ,N\right)}{1-\varpi_{r}^{Sh}\left(\mu ,N\right)}{\left(N_{r}⊗N_{s}\right)}^{p}\right)}^{\frac{1}{2p}}$$

## 3 Decision making mechanism of NC-SNWBM

For exploration of innovation and sustainability, decision making has a vital role in ambiguous and uncertain environment. Effective DM promotes proper management changes, collaboration and team work in remaining competitive, making good choices. For well informed decision in uncertain situation the flexible variables and designing scenarios are needed. Considering this a DM approach is design to meet the goal.

### 3.1 Construction of decision matrix

A decision matrix is a systematic and practical tool for making well informed decision in complex framework. It helps to identify the best alternative in objective manner by considering different criteria with their relative importance. The decision matrix consists of NC value subjected to criteria.

### 3.2 Determination of criterion weight

The role of weights in decision making play vital role in decision making theory. The weight can significantly change the results if the weight not properly assigned. Keeping in view this the Shapley measures are used to compute the weights.

### 3.3 Aggregated matrix

The NC-NSWBM operator is used to obtain an aggregated matrix streamlines decision making by assimilating different criteria subject to their relative importance. This approach enhances objectivity, well informed and balanced decision making.

### 3.4 Alternative ranking

**Fig. 2:**
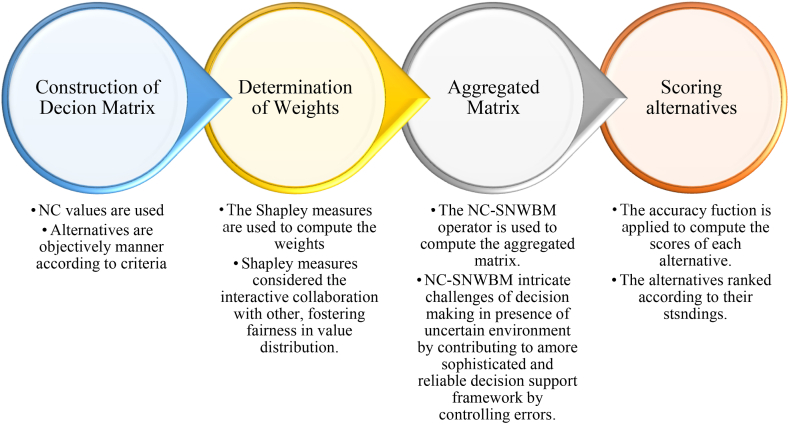
The working mechanism of proposed NC-SNWBM.

For comparison thee score functions are compute of a NC values. Based on these scored the alternatives are ranked so that the best choice can be considered**.** Fig. 1 **must be inserted before Introduction after the key words as Organization of manuscript.** The Fig. 1 presents the organization of manuscript. Flow chart (Fig. 2) is at the end of section 3. Decision making mechanism of NC-SNWBM (before 5. Application)

### 3.5 Flow chart

The flow chart provides the working mechanism of proposed methodology described in Fig. 2.

## 4 Application

This section consists of case study of DM problem exploring landscape of investment, sensitive analysis of parameters involved in proposed method, comparative analysis with some pre-existing literature to check validity of proposed method, recommendation based, advantages and conclusion.

### 4.1 Navigating investment: uncertainty to growth

A company's economic decision-making process is broad and involves a number of aspects, such as pricing, investments, market analysis, regulatory compliance, risk management, resource allocation, production, cost management, and sustainability. To navigate the complicated economic landscape and achieve long-term success, an enterprise requires a well-informed and strategic decision-making process.

The NC-SNWBM is evaluated using the example [17] that follows. To increase its overseas investment, a company must select the best country to invest in from a list of five options $\overset{⌢}{T}=\left\{{\overset{⌢}{l}}_{1},{\overset{⌢}{l}}_{2},{\overset{⌢}{l}}_{3},{\overset{⌢}{l}}_{4},{\overset{⌢}{l}}_{5}\right\}$. The economy, policy, infrastructure, and resources are the four primary variables (criteria) $E=\left\{e_{1},e_{2},e_{3},e_{4}\right\}$ that need to be considered. The NC data is presented below:

$$\left\{\overset{e_{1}}{\overset{︷}{\begin{array}{c} {℘}_{11}=\left(\left[0.700,0.800\right],\left[0.500,0.700\right],\left[0.100,0.200\right],0.900,0.700,0.200\right)\\ {℘}_{12}=\left(\left[0.600,0.800\right],\left[0.400,0.600\right],\left[0.400,0.600\right],0.200,0.300,0.700\right)\\ {℘}_{13}=\left(\left[0.400,0.500\right],\left[0.500,0.600\right],\left[0.400,0.600\right],0.300,0.300,0.300\right)\\ {℘}_{14}=\left(\left[0.400,0.500\right],\left[0.500,0.600\right],\left[0.400,0.500\right],0.500,0.400,0.500\right)\\ {℘}_{15}=\left(\left[0.600,0.700\right],\left[0.400,0.500\right],\left[0.400,0.500\right],0.500,0.400,0.500\right) \end{array}}}\right\}$$

$$\left\{\overset{e_{2}}{\overset{︷}{\begin{array}{c} {℘}_{21}=\left(\left[0.600,0.800\right],\left[0.400,0.500\right],\left[0.300,0.300\right],0.900,0.800,0.200\right)\\ {℘}_{22}=\left(\left[0.500,0.700\right],\left[0.400,0.600\right],\left[0.100,0.300\right],0.600,0.400,0.200\right)\\ {℘}_{23}=\left(\left[0.600,0.700\right],\left[0.400,0.600\right],\left[0.300,0.400\right],0.800,0.700,0.500\right)\\ {℘}_{24}=\left(\left[0.500,0.600\right],\left[0.300,0.400\right],\left[0.400,0.500\right],0.400,0.400,0.300\right)\\ {℘}_{25}=\left(\left[0.800,0.900\right],\left[0.300,0.400\right],\left[0.100,0.200\right],0.700,0.400,0.300\right) \end{array}}}\right\}$$

$$\left\{\overset{e_{3}}{\overset{︷}{\begin{array}{c} {℘}_{31}=\left(\left[0.800,0.800\right],\left[0.400,0.600\right],\left[0.100,0.200\right],0.800,0.900,0.100\right)\\ {℘}_{32}=\left(\left[0.600,0.600\right],\left[0.200,0.300\right],\left[0.400,0.500\right],0.500,0.100,0.300\right)\\ {℘}_{33}=\left(\left[0.700,0.800\right],\left[0.600,0.700\right],\left[0.100,0.200\right],0.500,0.400,0.400\right)\\ {℘}_{34}=\left(\left[0.600,0.800\right],\left[0.500,0.600\right],\left[0.100,0.200\right],0.500,0.700,0.300\right)\\ {℘}_{35}=\left(\left[0.700,0.800\right],\left[0.500,0.600\right],\left[0.100,0.200\right],0.500,0.300,0.400\right) \end{array}}}\right\}$$

$$\left\{\overset{e_{4}}{\overset{︷}{\begin{array}{c} {℘}_{41}=\left(\left[0.700,0.700\right],\left[0.300,0.400\right],\left[0.200,0.200\right],0.800,0.600,0.200\right)\\ {℘}_{42}=\left(\left[0.600,0.800\right],\left[0.400,0.400\right],\left[0.200,0.400\right],0.700,0.300,0.500\right)\\ {℘}_{43}=\left(\left[0.500,0.600\right],\left[0.500,0.600\right],\left[0.200,0.300\right],0.600,0.600,0.400\right)\\ {℘}_{44}=\left(\left[0.800,0.900\right],\left[0.300,0.400\right],\left[0.100,0.200\right],0.700,0.500,0.400\right)\\ {℘}_{45}=\left(\left[0.500,0.700\right],\left[0.500,0.500\right],\left[0.200,0.300\right],0.600,0.500,0.300\right) \end{array}}}\right\}$$

The interactive weights are obtained by fuzzy Shapley measure in Table 2.

The weights are calculated using SFM. For $\varpi_{1}^{Sh}$,. using equation (11), n=4, u=1, the set V in which $e_{1}$ is absent are considered from Table 3, total elements, v=0,1,2,3, respectively for collation empty, singleton, two elements and three elements.

$$\varpi_{1}^{Sh}=\frac{\left(4-1-0\right)!0!}{\left(4-1+1\right)!}\left(\left(\theta_{\lambda }\left(\left\{e_{1}\right\}\cup \phi \right)-\theta_{\lambda }\left(\phi \right)\right)\right)+\frac{\left(4-1-1\right)!1!}{\left(4-1+1\right)!}\left(\left(\theta_{\lambda }\left(\left\{e_{1}\right\}\cup \left\{e_{2}\right\}\right)-\theta_{\lambda }\left(\left\{e_{2}\right\}\right)\right)\right)+\frac{\left(4-1-1\right)!1!}{\left(4-1+1\right)!}\left(\left(\theta_{\lambda }\left(\left\{e_{1}\right\}\cup \left\{e_{3}\right\}\right)-\theta_{\lambda }\left(\left\{e_{3}\right\}\right)\right)\right)+\frac{\left(4-1-1\right)!1!}{\left(4-1+1\right)!}\left(\left(\theta_{\lambda }\left(\left\{e_{1}\right\}\cup \left\{e_{4}\right\}\right)-\theta_{\lambda }\left(\left\{e_{4}\right\}\right)\right)\right)+\frac{\left(4-1-2\right)!2!}{\left(4-1+1\right)!}\left(\left(\theta_{\lambda }\left(\left\{e_{1},e_{2},e_{3}\right\}\cup \left\{e_{2},e_{3}\right\}\right)-\theta_{\lambda }\left(\left\{e_{2},e_{3}\right\}\right)\right)\right)+\frac{\left(4-1-2\right)!2!}{\left(4-1+1\right)!}\left(\left(\theta_{\lambda }\left(\left\{e_{1},e_{2},e_{4}\right\}\cup \left\{e_{2},e_{4}\right\}\right)-\theta_{\lambda }\left(\left\{e_{2},e_{4}\right\}\right)\right)\right)+\frac{\left(4-1-2\right)!2!}{\left(4-1+1\right)!}\left(\left(\theta_{\lambda }\left(\left\{e_{1},e_{3},e_{4}\right\}\cup \left\{e_{3},e_{4}\right\}\right)-\theta_{\lambda }\left(\left\{e_{3},e_{4}\right\}\right)\right)\right)+\frac{\left(4-1-3\right)!3!}{\left(4-1+1\right)!}\left(\left(\theta_{\lambda }\left(\left\{e_{1},e_{2},e_{3},e_{4}\right\}\cup \left\{e_{2},e_{3},e_{4}\right\}\right)-\theta_{\lambda }\left(\left\{e_{2},e_{3},e_{4}\right\}\right)\right)\right)$$

**Table 3:** The data of interactive criteria along with their fuzzy measures.

A	$\mu_{\lambda }\left(A\right)$	A	$\mu_{\lambda }\left(A\right)$	A	$\mu_{\lambda }\left(A\right)$
ϕ	0.000	$\left\{e_{1}\right\}$	0.664	$\left\{e_{2}\right\}$	0.078
$\left\{e_{3}\right\}$	0.593	$\left\{e_{4}\right\}$	0.260	$\left\{e_{1},e_{2}\right\}$	0.144
$\left\{e_{1},e_{3}\right\}$	0.659	$\left\{e_{1},e_{4}\right\}$	0.327	$\left\{e_{2},e_{3}\right\}$	0.671
$\left\{e_{2},e_{4}\right\}$	0.338	$\left\{e_{3},e_{4}\right\}$	0.858	$\left\{e_{1},e_{2},e_{3}\right\}$	0.738
$\left\{e_{1},e_{2},e_{4}\right\}$	0.405	$\left\{e_{1},e_{3},e_{4}\right\}$	0.922	$\left\{e_{2},e_{3},e_{4}\right\}$	0.993
E	1.000

**Table 4:** The table for different value of p,q.

Values of p,q	Alternatives					Ranking
	A1	A2	A3	A4	A5	
p = 1,q = 1	0.585	0.552	0.572	0.579	0.573	A1>A4>A5>A3>A2
p = 1,q = 0	0.503	0.421	0.458	0.472	0.452	A1>A4>A3>A5>A2
p = 2,q = 0	0.586	0.538	0.570	0.579	0.559	A1>A4>A3>A5>A2
p = 0,q = 1	0.498	0.446	0.442	0.474	0.456	A1>A4>A5>A3>A2
p = 0,q = 2	0.587	0.565	0.560	0.585	0.579	A1>A4>A5>A2>A3
p = 0.3,q = 0	0.314	0.243	0.300	0.297	0.292	A1>A3>A4>A5>A2
p = 0.9,q = 0	0.424	0.335	0.379	0.390	0.375	A1>A4>A3>A5>A2
p = 0.6,q = 0	0.488	0.403	0.441	0.455	0.436	A1>A4>A3>A5>A2
p = 0,q = 0.3	0.317	0.266	0.276	0.297	0.275	A1>A4>A3>A5>A2
p = 0,q = 0.6	0.421	0.359	0.359	0.390	0.366	A1>A4>A5>A3 = A2
p = 0,q = 0.9	0.482	0.428	0.424	0.456	0.437	A1>A4>A5>A2>A3

**Fig. 3:**
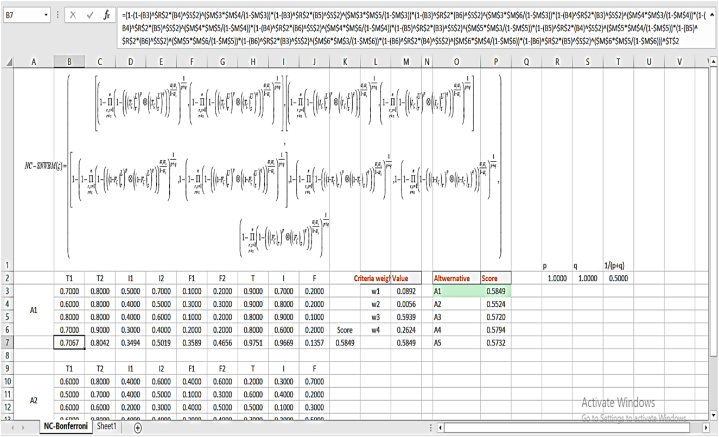
Excel spread sheet of computation.

The similar calculations yield the remaining weights. The overall weights of criteria are measured. $\varpi_{1}^{Sh}=0.0892,\varpi_{2}^{Sh}=0.0056,\varpi_{3}^{Sh}=0.5939,\varpi_{4}^{Sh}=0.2624\text{.}$ The NC-SNWBM is calculated for different values of p and q. Fig. 3 presents the coding in excel sheets.

The NC-SNWBM is evaluated for different values of p and q, the values and ranking of alternatives are tabulated below.

**Fig. 4:**
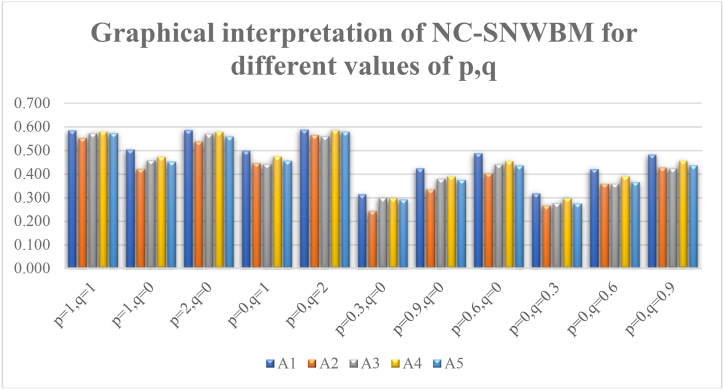
Graphical presentation of NC-SNWBM operator for different values of p,q near 1.

Fig. 4 provides the graphical view of NC-SNWBM for different values of p and q.

### 4.2 Sensitivity analysis

Sensitivity analysis is essential for comprehending how changes in parameters p,q may affect the dependability and consistency of the NC-SMWBM approach and identifying the regions where modifying the parameters may enhance the outcomes. Sensitivity analysis is used in conjunction with the NC-SNWBM approach and how modifications to certain factors impact the outcomes are discussed. This makes it possible to focus and resource on improving these crucial variables parameters, guaranteeing a more precise and trustworthy implementation of the NC-SMWBM approach. For deep analysis the calculation performed for different values of (p,q) the findings are as under (see Table 4).

**Fig. 5:**
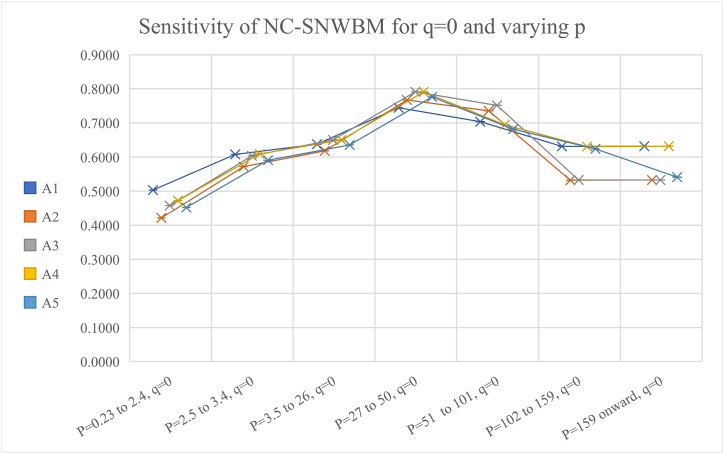
Graphical presentation of NC-SNWBM operator for different values of p and keeping q=0.

Table 5 and Fig. 5 presents the impact of keeping q = 0 and p varying; it can be observed that overall, the results are sensitive. But for smaller q near 1 and large value of p more than 100, the results agree. So, one must be careful assigning values to p and q.

**Table 5:** The rankings, keeping q = 0 and varying p.

Values of p,q	Ranking
P = 0.23 to 2.4, q = 0	A1>A4>A3>A5>A2
P = 2.5 to 3.4, q = 0	A4>A1>A3>A5>A2
P = 3.5 to 26, q = 0	A5>A4>A1>A5>A2
P = 27 to 50, q = 0	A4>A3>A5>A2>A1
P = 51 to 101, q = 0	A3>A2>A1>A4>A5
P = 102 to 159, q = 0	A1>A3>A5>A4>A2
P = 159 onward, q = 0	A1 = A4>A3>A5 = A2

**Fig. 6:**
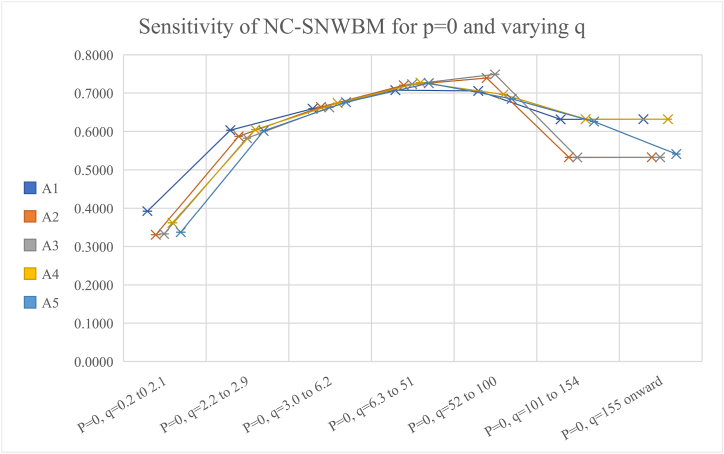
Graphical presentation of NC-SNWBM operator for different values of q and keeping p = 0.

Table 6 and Fig. 6 present the sensitivity by keeping p = 0 and p is varying, it can be observed that for smaller value of q near 1 and large value of p more than 101, the results agree. So, one must be careful assigning values to p and q.

**Table 6:** The rankings, keeping p = 0 and varying q.

Values of p,q	Ranking
P = 0, q = 0.2 to 2.1	A1>A4>A5>A3>A2
P = 0, q = 2.2 to 2.9	A4>A1>A5>A2>A3
P = 0, q = 3.0 to 6.2	A5>A4>A2>A4>A1
P = 0, q = 6.3 to 51	A4>A5>A3>A2>A1
P = 0, q = 52 to 100	A3>A2>A1>A4>A5
P = 0, q = 101 to 154	A4>A1>A5>A3>A2
P = 159 onward, q = 0	A1 = A4>A5>A3 = A2

**Fig. 7:**
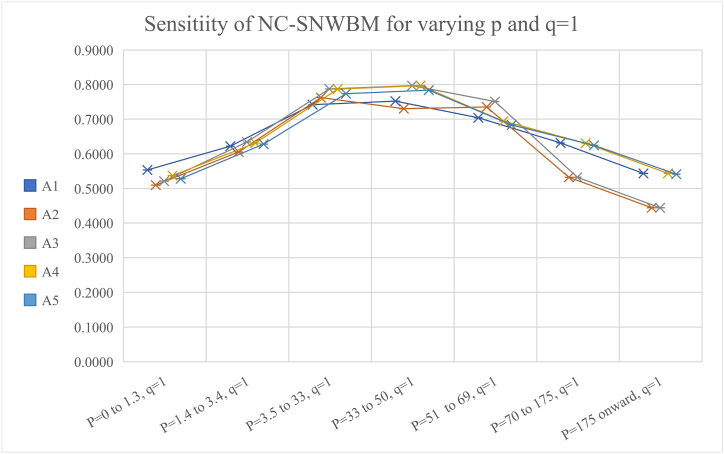
Graphical presentation of NC-SNWBM operator for different values of p and keeping q=1.

Table 7 and Fig. 7 present the sensitivity by keeping q = 1 and p is varying, it can be observed that for smaller value of q near 1 and large value of p more than 70, the results agrees. So, one must be careful assigning values to p and q.

**Table 7:** The rankings, keeping q = 1 and varying p.

Values of p,q	Ranking
P = 0 to 1.3, q = 1	A1>A4>A5>A3>A2
P = 1.4 to 3.4, q = 1	A3>A4>A5>A1>A2
P = 3.5 to 33, q = 1	A3>A4>A5>A1>A2
P = 33 to 50, q = 1	A4>A3>A5>A1>A2
P = 51 to 69, q = 1	A3>A2>A1>A4>A5
P = 70 to 175, q = 1	A1>A3>A5>A3>A2
P = 175 onward, q = 1	A1 = A4>A5>A3 = A2

Form table and figure it can be observed that the NC-SNWBM is sensitive for p and. The brief summary is presented it Table 8 below.

**Table 8:** The impact of p,q to over alternative.

P	q	Best alternative
0.23 to 2.4	0	A1
2.5 to 3.4	0	A4
3.5 to 26	0	A3
27 to 50	0	A4
51 to 101	0	A3
102 to 159	0	A1
160 onward	0	Both A1 and A4
0	0.2to2.1	A1
0	2.2 to 2.9	A4
0	3.0 to 6.2	A5
0	6.3 to 51	A4
0	52 to 100	A3
0	101 to 154	A4
0	155 onward	Both A1 and A4
1	1	A1
2 to 31	1	A3
33 to 44	1	A4
35 to 44	1	A4
45 to 69	1	A3
70 to 175	1	A1
176 onward	1	Both A1 and A4

It can be observed that, for either of the two parameters p,q, the BM approach produced different alternative as best in certain ranges. Considering q = 0 and ranges p between 0 and 2.4, more than 100, p = 0 and q ranges 0.2 to 2.1, more 160, the A1 is best choice. Considering q = 0 and ranges p between 2.4 and 3.4, 27 to 50 more than 155, p = 0 and q ranges 2.2 to 2.9, more 155, the A4 is best choice. Considering q = 0 and ranges p between 2.5 and 26, 51 to 101, p = 0 and q ranges 52 to 100, the A3 is the best choice. Different range of parameters has different choices. This indicates that these parameters have a substantial effect on the outcome.

The observations are evident that the variation in p,q values within a suitable range affects the outcome in NC-SNWBM. Through this research can evaluate the impact of changing the parameters being used on the BM method's overall efficacy and the range of values that may be achieved.

To decide the suitable range for parameters p q, the comparative analysis of the proposed method is done with some existing methods.

### 4.3 Comparative analysis

If the parameters used in the method are sensitive, it is important to perform a comparative analysis. It provides insight into the impact of parameter variations on method performance, aids in optimization, and identifies and establishes method reliability and validity. Such analysis allows one to make more informed decisions, increases the reliability of methodology, and ensures its effectiveness in providing accurate and reliable results. The comparison of NC-SNWBM with some following MCDM methods in NCS environment is tabulated in Table 9.

**Table 9:** The tabulated view of comparative analysis.

Methodology	Ranking	Ref.
NC Einstein geometric aggregation [27]	A1>A3>A2>A5>A4	[27]
Generalized NC aggregation [29]	A1>A3>A5>A4>A2	[25]
GRA method in NCS [32]	A1>A4>A3>A5>A2	[28]
NC Cosine measure [33]	A1>A4>A3>A5>A2	[29]
IGNCSCA [58]	A1>A3>A4>A5>A2	[54]
NC-CODAS [58]	A1>A3>A4>A5>A2	[54]

**NC Einstein geometric aggregation** is the generalization of geometric aggregation operator based on Einstein norms [27].

**Generalized NC aggregation** consist of a bunch of generation like averaging, geometric hybrid aggregation operators in NCS environment [29].

**GRA method** [32]A Grey rational analysis technique used in DM under NCS Banerjee et al., [32].

**Cosine similarity measures** used in DM over NCS environment by Lu and Ye [33].

**NC-CODAS** consist of induced generalized averaging and geometric aggregation operators NCS and CODAS method is applied in DM by Majid et al., [58].

From Table 9, it is observed that A1 is the best and A2 the worst alternative computed by the methods except the first method in the table.

### 4.4 Recommendations

In view of sensitivity and comparative analysis, the following recommendations are proposed.

Focus on refining the two parameters p,q that have the highest sensitivity according to the NS-SNWBM method. The reliability and accuracy of these results are significantly affected by these parameters that have a sensitivity.

To understand the behavior and effects of these parameters more thoroughly, it is recommended that target research and testing be conducted in order to enhance the performance of the NS-SNWBM method. To obtain a better understanding of their effects, it will be possible to make informed decisions on how they should be adjusted and optimized for the given range.

Moreover, in order to evaluate the reliability of this model, it is important that a Nc-SNWBM method be periodically tested with different values for these parameters. This will help to maintain the reliability and consistency of the NS-SNWBM method in a variety of scenarios and conditions.

Finally, the efficiency and applicability of BM can be enhanced by taking a closer look at influential parameters p,q which have been identified from sensitivity analysis and constantly fine tuning their values. This will lead to more precise and robust outcomes, giving valuable insight into the way decisions are made.

### 4.5 Advantages of NC-SNWBM

The NS-SNWBM is a generalization of many aggregation operators. The parameters provide a vast range that can the decision makers and Straight forward methodology to evaluate. From comparative analysis, it is observed that the ranking is influenced by different sets, parameters, and geometrically based operators. However, the NC-SNWBM is still an efficient method for problems dealing with the weight of decision-makers and their overall interactions. It also has the characteristic of managing interaction between inputs and efficiently managing errors. Unlike traditional linear aggregation methods (e.g., weighted averages), the NC-SNWBM allows for the modeling of dependencies and interactions among criteria. NC-SNWBM can cope with situations where the significance of one criterion depends on the presence or absence of others. Furthermore, the NCS provides the environment to handle membership, non-membership, and indeterminacy in both interval and crisp form, easing the decision-maker making choices.

## 5 Conclusion

The versatility of the NC-SNWBM method extends its applicability across a spectrum of domains. Whether applied in decision support systems, artificial intelligence, or areas requiring information fusion, the method offers a robust and flexible tool for aggregating information. The integration of NS, Shapley values, and the BM aggregation operator provides a holistic framework for handling uncertainty, imprecision, and collaboration in DM. In a company, the effective DM always depends on investment, and cost management keeping risk and sustainability in view. A strategic and well-informed DM process is needed to counter the complex economic landscape and achieve long term success. The proposed method involves as well weighted marginal contribution of contributors to encounter these factors significantly. Further, the weights are measured by Shapley and aggregated by BM in the NCS environment making it the optimal method. The method is compared with pre-existing methods to validate its validity Table 9. The key feature of NC-SNWBM is to control different types of error by properly handling the parameters p and q. A detailed view of impacting results with the variation of p and q is presented in Table 5and Fig. 5, Table 6 and Fig. 6, Table 7and Fig. 7, and Table 8 with recommendations. The future generalizations of NC-SNWBM such as Hamachar, Aczel-Alsina will be explored. The integration of other fuzzy measures like distance and trigonometric measures can be evaluated in NC and other extensions of the fuzzy set. Further, the proposed NC-SNWBM can be applied to numerical problems that have complex frames of environment like information fusion, artificial intelligence, decision support systems, risk analysis and energy requirement.

## Data availability

This study did not involve the use of any data.

## Funding

This Research is funded by Research Supporting Project Number (RSPD2024R553), 10.13039/501100002383King Saud University, and Riyadh, Saudi Arabia.

## CRediT authorship contribution statement

**Majid Khan:** Writing – review & editing, Writing – original draft. **Muhammad Gulistan:** Formal analysis, Conceptualization. **Musaed Alhussein:** Visualization, Validation, Investigation. **Khursheed Aurangzeb:** Methodology, Investigation. **Adnan Khurshid:** Supervision, Resources.

## Declaration of competing interest

The authors declare that they have no known competing financial interests or personal relationships that could have appeared to influence the work reported in this paper.

## Figures and Tables

**Figure:**
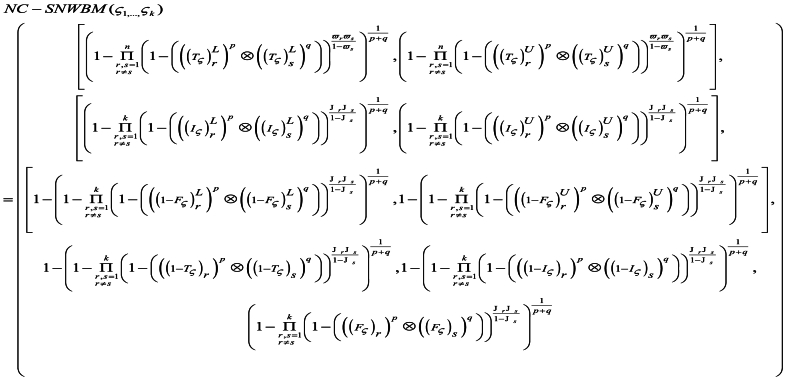


**Figure:**
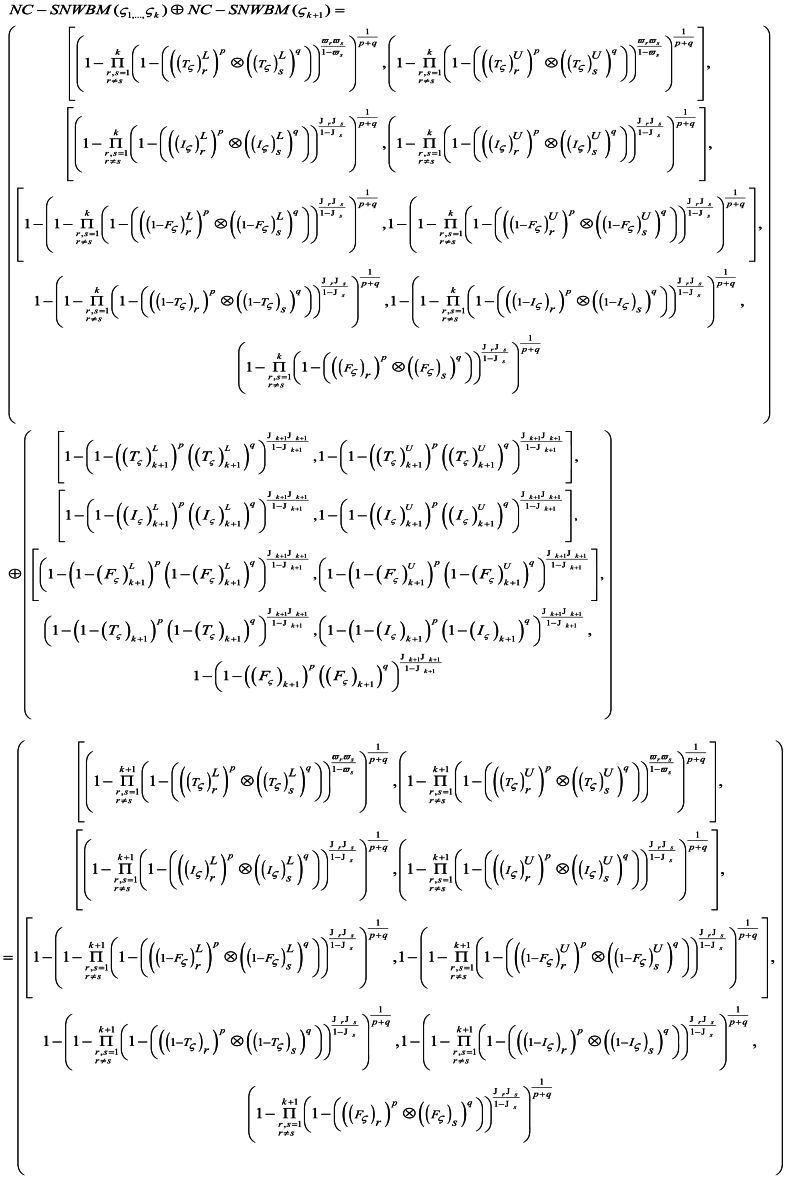


## Appendix

The proof of some theorems provided. Proof of Theorem 1 The theorem is proved by mathematical induction.

$$NC-SNWBM\left(\varsigma \right)={\left(\sum_{\begin{array}{c} r,s=1\\ r\neq s \end{array}}^{n}\frac{\varpi_{r}^{Sh}\left(\mu ,N\right)\varpi_{s}^{Sh}\left(\mu ,N\right)}{1-\varpi_{r}^{Sh}\left(\mu ,N\right)}\left({\left(\varsigma_{r}\right)}^{p}⊗{\left(\varsigma_{s}\right)}^{q}\right)\right)}^{\frac{1}{p+q}}$$

For convenience let $\varpi_{r}^{Sh}\left(\mu ,N\right)={℧}_{r}$, so

$$NC-SNWBM\left(\varsigma \right)={\left(\sum_{\begin{array}{c} r,s=1\\ r\neq s \end{array}}^{n}\frac{\varpi_{r}^{Sh}\left(\mu ,N\right)\varpi_{s}^{Sh}\left(\mu ,N\right)}{1-\varpi_{r}^{Sh}\left(\mu ,N\right)}\left({\left(\varsigma_{r}\right)}^{p}⊗{\left(\varsigma_{s}\right)}^{q}\right)\right)}^{\frac{1}{p+q}}={\left(\sum_{\begin{array}{c} r,s=1\\ r\neq s \end{array}}^{n}\frac{{℧}_{r}{℧}_{s}}{1-{℧}_{r}}\left({\left(\varsigma_{r}\right)}^{p}⊗{\left(\varsigma_{s}\right)}^{q}\right)\right)}^{\frac{1}{p+q}}$$

By operation laws,

$${\left(\varsigma_{r}\right)}^{p}=\left(\left[{\left({\left(T_{\varsigma }\right)}_{r}^{L}\right)}^{p},{\left({\left(T_{\varsigma }\right)}_{r}^{U}\right)}^{p}\right],\left[{\left({\left(I_{\varsigma }\right)}_{r}^{L}\right)}^{p},{\left({\left(I_{\varsigma }\right)}_{r}^{U}\right)}^{p}\right],\left[1-{\left(1-{\left(F_{\varsigma }\right)}_{r}^{L}\right)}^{p},1-{\left(1-{\left(F_{\varsigma }\right)}_{r}^{U}\right)}^{p}\right],1-{\left(1-{\left(T_{\varsigma }\right)}_{r}\right)}^{p},1-{\left(1-{\left(I_{\varsigma }\right)}_{r}\right)}^{p},{\left({\left(F_{\varsigma }\right)}_{r}\right)}^{p}\right)$$

$${\left(\varsigma_{s}\right)}^{q}=\left(\left[{\left({\left(T_{\varsigma }\right)}_{s}^{L}\right)}^{q},{\left({\left(T_{\varsigma }\right)}_{s}^{U}\right)}^{q}\right],\left[{\left({\left(I_{\varsigma }\right)}_{s}^{L}\right)}^{q},{\left({\left(I_{\varsigma }\right)}_{s}^{U}\right)}^{q}\right],\left[1-{\left(1-{\left(F_{\varsigma }\right)}_{s}^{L}\right)}^{q},1-{\left(1-{\left(F_{\varsigma }\right)}_{s}^{U}\right)}^{q}\right],1-{\left(1-{\left(T_{\varsigma }\right)}_{s}\right)}^{q},1-{\left(1-{\left(I_{\varsigma }\right)}_{s}\right)}^{q},{\left({\left(F_{\varsigma }\right)}_{s}\right)}^{q}\right)$$

$${\left(\varsigma_{r}\right)}^{p}⊗{\left(\varsigma_{s}\right)}^{q}=\left(\begin{array}{c} \left[{\left({\left(T_{\varsigma }\right)}_{r}^{L}\right)}^{p}{\left({\left(T_{\varsigma }\right)}_{s}^{L}\right)}^{q},{\left({\left(T_{\varsigma }\right)}_{r}^{U}\right)}^{p}{\left({\left(T_{\varsigma }\right)}_{s}^{U}\right)}^{q}\right],\left[{\left({\left(I_{\varsigma }\right)}_{r}^{L}\right)}^{p}{\left({\left(I_{\varsigma }\right)}_{s}^{L}\right)}^{q},{\left({\left(I_{\varsigma }\right)}_{r}^{U}\right)}^{p}{\left({\left(I_{\varsigma }\right)}_{s}^{U}\right)}^{q}\right],\\ \left[1-{\left(1-{\left(F_{\varsigma }\right)}_{r}^{L}\right)}^{p}{\left(1-{\left(F_{\varsigma }\right)}_{s}^{L}\right)}^{q},1-{\left(1-{\left(F_{\varsigma }\right)}_{r}^{U}\right)}^{p}{\left(1-{\left(F_{\varsigma }\right)}_{s}^{U}\right)}^{q}\right],\\ 1-{\left(1-{\left(T_{\varsigma }\right)}_{r}\right)}^{p}{\left(1-{\left(T_{\varsigma }\right)}_{s}\right)}^{q},1-{\left(1-{\left(I_{\varsigma }\right)}_{r}\right)}^{p}{\left(1-{\left(I_{\varsigma }\right)}_{s}\right)}^{q},{\left({\left(F_{\varsigma }\right)}_{r}\right)}^{p}{\left({\left(F_{\varsigma }\right)}_{s}\right)}^{q} \end{array}\right)$$

$$\frac{{℧}_{r}{℧}_{s}}{1-{℧}_{r}}\left({\left(\varsigma_{r}\right)}^{p}⊗{\left(\varsigma_{s}\right)}^{q}\right)=\left(\begin{array}{c} \left[1-{\left(1-{\left({\left(T_{\varsigma }\right)}_{r}^{L}\right)}^{p}{\left({\left(T_{\varsigma }\right)}_{s}^{L}\right)}^{q}\right)}^{\frac{{℧}_{r}{℧}_{s}}{1-{℧}_{r}}},1-{\left(1-{\left({\left(T_{\varsigma }\right)}_{r}^{U}\right)}^{p}{\left({\left(T_{\varsigma }\right)}_{s}^{U}\right)}^{q}\right)}^{\frac{{℧}_{r}{℧}_{s}}{1-{℧}_{r}}}\right],\left[1-{\left(1-{\left({\left(I_{\varsigma }\right)}_{r}^{L}\right)}^{p}{\left({\left(I_{\varsigma }\right)}_{s}^{L}\right)}^{q}\right)}^{\frac{{℧}_{r}{℧}_{s}}{1-{℧}_{r}}},1-{\left(1-{\left({\left(I_{\varsigma }\right)}_{r}^{U}\right)}^{p}{\left({\left(I_{\varsigma }\right)}_{s}^{U}\right)}^{q}\right)}^{\frac{{℧}_{r}{℧}_{s}}{1-{℧}_{r}}}\right],\\ \left[{\left(1-{\left(1-{\left(F_{\varsigma }\right)}_{r}^{L}\right)}^{p}{\left(1-{\left(F_{\varsigma }\right)}_{s}^{L}\right)}^{q}\right)}^{\frac{{℧}_{r}{℧}_{s}}{1-{℧}_{r}}},{\left(1-{\left(1-{\left(F_{\varsigma }\right)}_{r}^{U}\right)}^{p}{\left(1-{\left(F_{\varsigma }\right)}_{s}^{U}\right)}^{q}\right)}^{\frac{{℧}_{r}{℧}_{s}}{1-{℧}_{r}}}\right],{\left(1-{\left(1-{\left(T_{\varsigma }\right)}_{r}\right)}^{p}{\left(1-{\left(T_{\varsigma }\right)}_{s}\right)}^{q}\right)}^{\frac{{℧}_{r}{℧}_{s}}{1-{℧}_{r}}},{\left(1-{\left(1-{\left(I_{\varsigma }\right)}_{r}\right)}^{p}{\left(1-{\left(I_{\varsigma }\right)}_{s}\right)}^{q}\right)}^{\frac{{℧}_{r}{℧}_{s}}{1-{℧}_{r}}},\\ 1-{\left(1-{\left({\left(F_{\varsigma }\right)}_{r}\right)}^{p}{\left({\left(F_{\varsigma }\right)}_{s}\right)}^{q}\right)}^{\frac{{℧}_{r}{℧}_{s}}{1-{℧}_{r}}} \end{array}\right)$$

Let the condition hold good for n=k, Proof of theorem 2

$$\left(\text{Reducibility}\right)\;\text{Let}\;\varpi_{r}\left(\mu ,N\right)=\left(\frac{1}{n},\frac{1}{n},\ldots ,\frac{1}{n}\right)\text{,}$$

$$NC-SNWBM\left(\varsigma \right)={\left(\sum_{\begin{array}{c} r,s=1\\ r\neq s \end{array}}^{n}\frac{\varpi_{r}^{Sh}\left(\mu ,N\right)\varpi_{s}^{Sh}\left(\mu ,N\right)}{1-\varpi_{r}^{Sh}\left(\mu ,N\right)}\left({\left(\varsigma_{r}\right)}^{p}⊗{\left(\varsigma_{s}\right)}^{q}\right)\right)}^{\frac{1}{p+q}}={\left(\sum_{\begin{array}{c} r,s=1\\ r\neq s \end{array}}^{n}\frac{\frac{1}{n}\frac{1}{n}}{1-\frac{1}{n}}\left({\left(\varsigma_{r}\right)}^{p}⊗{\left(\varsigma_{s}\right)}^{q}\right)\right)}^{\frac{1}{p+q}}={\left(\sum_{\begin{array}{c} r,s=1\\ r\neq s \end{array}}^{n}\frac{1}{n\left(n-1\right)}\left({\left(\varsigma_{r}\right)}^{p}⊗{\left(\varsigma_{s}\right)}^{q}\right)\right)}^{\frac{1}{p+q}}=NC-BM\left(\varsigma_{r}\right)$$

Proof of theorem 3

$$\left(\text{Idempotency}\right)\;NC-SNWBM\left(\varsigma_{r}\right)={\left(\sum_{\begin{array}{c} r,s=1\\ r\neq s \end{array}}^{n}\frac{\varpi_{r}^{Sh}\left(\mu ,N\right)\varpi_{s}^{Sh}\left(\mu ,N\right)}{1-\varpi_{r}^{Sh}\left(\mu ,N\right)}\left({\left(\varsigma_{r}\right)}^{p}⊗{\left(\varsigma_{s}\right)}^{q}\right)\right)}^{\frac{1}{p+q}}={\left(\sum_{\begin{array}{c} r,s=1\\ r\neq s \end{array}}^{n}\frac{\varpi_{r}^{Sh}\left(\mu ,N\right)\varpi_{s}^{Sh}\left(\mu ,N\right)}{1-\varpi_{r}^{Sh}\left(\mu ,N\right)}\left({\left(\varsigma_{r}\right)}^{p}⊗{\left(\varsigma_{r}\right)}^{q}\right)\right)}^{\frac{1}{p+q}}=\varsigma$$

Proof of t theorem 4 (Monotonicity) Consider the following inequalities: N,M∈(0,1), For ${\left(N\right)}^{k}\leq {\left(M\right)}^{k}\Rightarrow 1-{\left(N\right)}^{k}\geq 1-{\left(M\right)}^{k}\Rightarrow 1-\left(1-{\left(N\right)}^{k}\right)\leq 1-\left(1-{\left(M\right)}^{k}\right)\text{,}$ (i) and ${\left(N\right)}^{k}\geq {\left(M\right)}^{k}\Rightarrow 1-{\left(N\right)}^{k}\leq 1-{\left(M\right)}^{k}\Rightarrow 1-\left(1-{\left(N\right)}^{k}\right)\leq 1-\left(1-{\left(M\right)}^{k}\right)$ (ii) For N=M=0 and N=M=1 result is trivial. Putting NM≤1forallk. For $N={\left(T_{N}\right)}_{r}^{L},M={\left(T{*}_{N}\right)}_{s}^{L}\text{.}$ and k=p,q using equation (i),

$${\left(1-\overset{n}{\underset{\begin{array}{c} r,s=1\\ r\neq s \end{array}}{\Pi }}{\left(1-\left({\left({\left(T_{N}\right)}_{r}^{L}\right)}^{p}⊗{\left({\left(T_{M}\right)}_{s}^{L}\right)}^{q}\right)\right)}^{\frac{\varpi_{r}\varpi_{s}}{1-\varpi_{s}}}\right)}^{\frac{1}{p+q}}\leq {\left(1-\overset{n}{\underset{\begin{array}{c} r,s=1\\ r\neq s \end{array}}{\Pi }}{\left(1-\left({\left({\left({T_{N}}^{*}\right)}_{r}^{L}\right)}^{p}⊗{\left({\left(T_{M}\right)}_{s}^{L}\right)}^{q}\right)\right)}^{\frac{\varpi_{r}\varpi_{s}}{1-\varpi_{s}}}\right)}^{\frac{1}{p+q}}$$

The same result can be deduced for ${\left(T_{N}\right)}_{r}^{U},{\left(T{*}_{N}\right)}_{s}^{U},{\left(I_{N}\right)}_{r}^{L},{\left(I{*}_{N}\right)}_{s}^{L},{\left(I_{N}\right)}_{r}^{U},{\left(I{*}_{N}\right)}_{s}^{U},{\left(F_{N}\right)}_{r},{\left(F_{N}\right)}_{s}$. Using equation (ii),

$$1-{\left(1-\overset{n}{\underset{\begin{array}{c} r,s=1\\ r\neq s \end{array}}{\Pi }}{\left(1-\left({\left({\left(1-F_{\varsigma }\right)}_{r}^{L}\right)}^{p}⊗{\left({\left(1-F_{\varsigma }\right)}_{s}^{L}\right)}^{q}\right)\right)}^{\frac{\varpi_{r}\varpi_{s}}{1-\varpi_{s}}}\right)}^{\frac{1}{p+q}}\geq 1-{\left(1-\overset{n}{\underset{\begin{array}{c} r,s=1\\ r\neq s \end{array}}{\Pi }}{\left(1-\left({\left({\left(1-{F_{\varsigma }}^{*}\right)}_{r}^{L}\right)}^{p}⊗{\left({\left(1-F_{\varsigma }\right)}_{s}^{L}\right)}^{q}\right)\right)}^{\frac{\varpi_{r}\varpi_{s}}{1-\varpi_{s}}}\right)}^{\frac{1}{p+q}}\text{,}$$

Analogy for ${\left(F_{N}\right)}_{r}^{U},{\left(F{*}_{N}\right)}_{s}^{U},{\left(T_{N}\right)}_{r},{\left(T{*}_{N}\right)}_{s},{\left(I_{N}\right)}_{r},{\left(I_{N}\right)}_{s}$.

$$NC-SNWBM\left(N_{r}\right)\leq NC-SNWBM\left({N_{r}}^{*}\right)$$

Proof of theorem 5 (Boundedness), followed by theorem 4 and theorem 5.
